# Successful control of an environmental reservoir of NDM-producing *Klebsiella pneumoniae* associated with nosocomial transmissions in a low-incidence setting

**DOI:** 10.1186/s13756-024-01488-0

**Published:** 2024-10-29

**Authors:** Estelle Moulin, Paraskevas Filippidis, Corinne Aymon Paire-Ficout, Dominique S. Blanc, Bruno Grandbastien, Laurence Senn

**Affiliations:** 1 Infection Prevention and Control Unit, Infectious Diseases Service, Lausanne University Hospital, University of Lausanne, Lausanne, 1011 Switzerland; 2 Infectious Diseases Service, Lausanne University Hospital and University of Lausanne, Lausanne, Switzerland; 3 Swiss National Reference Center for Emerging Antibiotic Resistance, (NARA), University of Fribourg, Fribourg, Switzerland

**Keywords:** Nosocomial outbreak, Carbapenemase, NDM, Sink traps, Wastewater drains reservoir, Whole genome sequencing

## Abstract

**Background:**

The hospital wastewater system has been reported as a source of nosocomial acquisition of carbapenemase producing *Enterobacteriaceae* (CPE) in various settings. Cleaning and disinfection protocols or replacement of contaminated equipment often fail to eradicate these environmental reservoirs, which can lead to long-term transmission of CPE. We report a successful multimodal approach to control a New Delhi metallo-beta-lactamase positive *Klebsiella pneumoniae* (NDM-KP) nosocomial outbreak implicating contamination of sink traps in a low-incidence setting.

**Methods:**

Following the incidental identification of NDM-KP in a urine culture of an inpatient, we performed an epidemiological investigation, including patient and environmental CPE screening, and whole genome sequencing (WGS) of strains. We also implemented multimodal infection prevention and control (IPC) measures, namely the isolation of cases, waterless patient care, replacement of contaminated P-traps and connecting pieces, and bleach and steam disinfection of sinks for 6 months, followed by patient and environmental screenings for eradication.

**Results:**

Between February and May 2022, five NDM-KP cases were identified in an eight-bed neurosurgical intermediate care unit. Among the eight sink traps of the unit, three were positive for NDM-KP. Patient and environmental isolates belonged to multilocus sequence typing ST-268. All isolate genomes were genetically very similar suggesting cross-transmission and a potential role of the environment as the source of transmissions. Following the introduction of combined IPC measures, no new case was subsequently detected and sink traps remained negative for NDM-KP within 6 months after the intervention.

**Conclusion:**

The implementation of multimodal IPC measures, including waterless patient care combined with the replacement and disinfection of P-traps and connecting pieces, was successful in the control of NDM-KP after eight months. In a low-incidence setting, this approach has made it possible to pursue the objective of zero transmission of carbapenemase-producing *Enterobacteriaceae* (CPE).

## Introduction

Increasing incidence of carbapenemase-producing *Enterobacteriaceae* (CPE) poses a major public-health threat. Effective treatment of CPE infections is often delayed, with limited options available, leading to high mortality. Data from the European Antimicrobial Resistance Surveillance Network (EARS-Net) show large differences in the prevalence of CPE depending on the country [1]. In Switzerland, data from the Swiss Centre for Antibiotic Resistance (ANRESIS) indicate that carbapenem resistance, albeit still rare, is steadily increasing, reflecting trends in neighbouring countries [2]. Infection prevention and control (IPC) measures and the implementation of antimicrobial stewardship programs are crucial for preventing and controlling the emergence and spread of CPE.

The hospital wastewater system has been identified as a potential source of nosocomial acquisition of carbapenemase-producing *Pseudomonas* spp. or *Acinetobacter* spp, as well as CPE, in various settings [3–10]. Reported outbreaks were characterised by a low overall incidence of clinical cases and highly variable durations, likely due to differences in the speed of detection, the type of measures applied, and the duration of follow-up after their implementation [11]. Cleaning and disinfection protocols or replacement of contaminated equipment often fail to eradicate these environmental reservoirs, leading to long-term transmission of CPE [12–15]. 

We report a successful multimodal approach to control a nosocomial outbreak of New Delhi metallo-beta-lactamase positive *Klebsiella pneumoniae* (NDM-KP) associated with the contamination of sink traps in a neurosurgical intermediate care unit of a Swiss hospital.

## Methods

### Setting

**Fig. 1:**
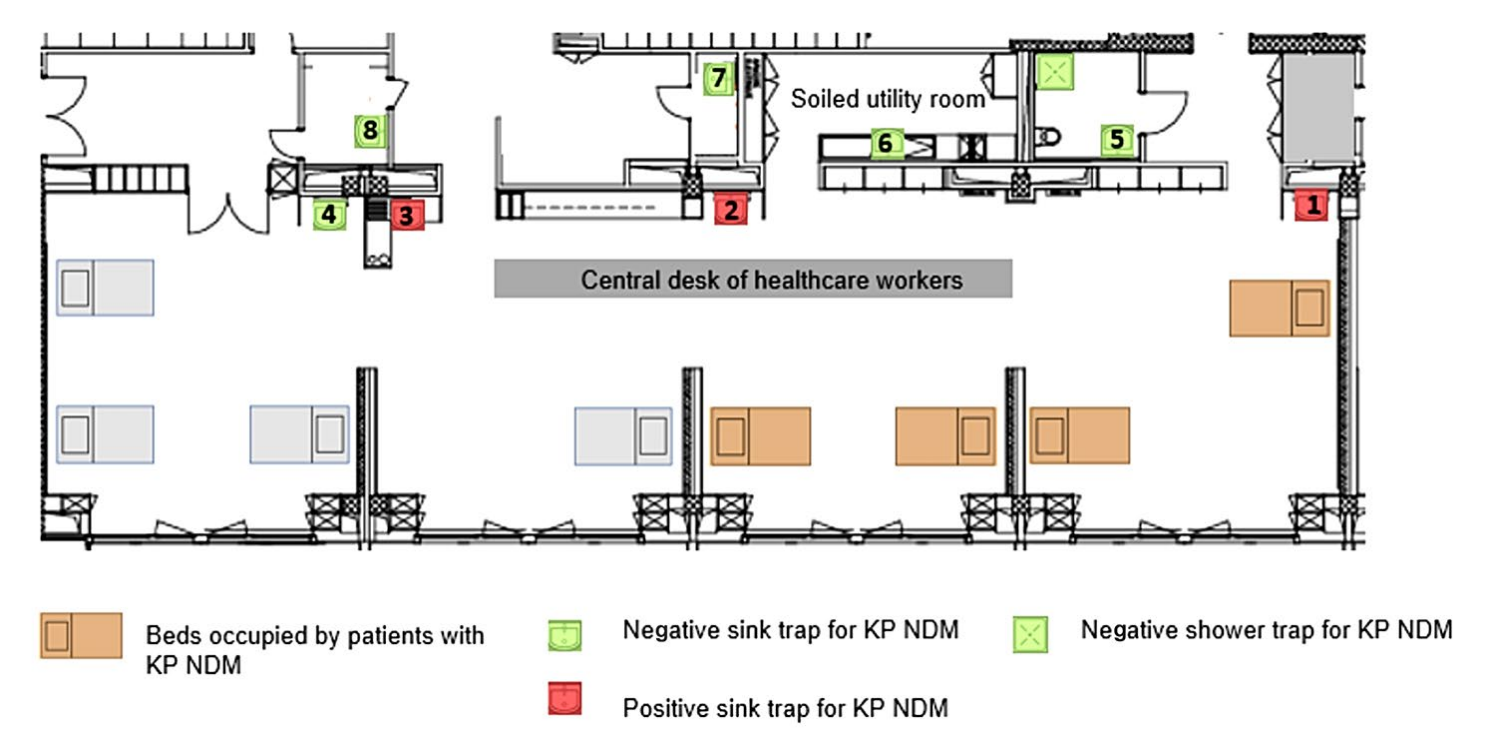
Map of the intermediate care unit including the location of positive patients and sink traps

Lausanne University Hospital (CHUV) is a 1100-bed teaching hospital serving as a primary-level community hospital for Lausanne (catchment population 300’000) and as a secondary and tertiary referral hospital for Western Switzerland (catchment population 1-1.5 million). Among the *Enterobacteriaceae* identified at CHUV, the prevalence of CPE remains less than 1% and the CPE incidence less than 1 case per 1000 admissions, mainly consisting of sporadic cases imported from abroad. The reported outbreak took place in an eight-bed neurosurgical intermediate care unit, including beds for severely brain-impaired patients. Beds are distributed in an open-space area with a central desk for healthcare workers and four sinks. Four additional sinks and one shower are located in adjacent rooms. The map of the unit is shown in Fig. 1.

### IPC measures and CPE control policy

After receiving automatic alerts for all patients colonised with multidrug resistance organisms (MDRO), the IPC team ensures that contact isolation measures are in place and carries out investigations of contact patients. Weekly screening for intestinal carriage of MDRO, including CPE, is systematically performed among all intensive care unit (ICU) patients. In non-ICU units, targeted individual screening is performed at admission, based on conventional risk factors such as previous carriage, contact with a positive case (sharing a room or an open space unit with a positive patient before isolation was introduced) or recent hospitalisation abroad [16]. According to the Swiss national IPC guidelines [17], when a new CPE-positive case is identified, contact isolation of the positive patient and its contacts is promptly applied. CPE screening of the contacts on rectal swabs is performed on days 0, 7 and 14 (RT-PCR and culture on day 0, culture only on day 7 and 14). Moreover, chlorhexidine bathing of positive cases is recommended during their hospital stays.

For the reported outbreak, following the identification of a NDM-KP in a urine culture from a patient hospitalised in the neurosurgical intermediate care unit, all the aforementioned epidemiological measures were implemented. Contact precautions were maintained for CPE contact patients until they tested negative on three consecutive weekly screenings. In addition to contacts screening, weekly CPE screenings of all patients were performed on a weekly basis (and then every two weeks) due to the open space architecture of the unit. This continued until discharge of the last positive case, for six months. The IPC team carried out observations of practices and educational rounds to reinforce hand hygiene, material and environment disinfection, and adherence to aseptic care procedures.

### Environmental sampling

Environmental sampling for CPE from sink traps (P-trap and connecting pieces) and the shower drain was conducted between May 2022 and May 2023, with a total of 114 samples collected: 102 from eight sink traps and 12 from the shower drain. P-traps samples were collected using eSwab^®^ (Copan, Italy). The swab was attached to a metal rod before being inserted through the drain into the siphon. Ten back-and-forth motions against the siphon walls were performed before breaking the swab into the eSwab tube containing Amies liquid.

### Bacterial identification, molecular characterization, and genomic analysis

Patients and environmental samples were inoculated on selective SuperCarba medium (CHROMagar™, France), and incubated at 37 °C for 24 h. Suspected colonies were identified with MALDI-TOF (Bruker, Germany). The presence of carbapenemase was confirmed using the lateral flow immunoassay NG-Test^®^ CARBA-5 (NG Biotech, France). In parallel to culture, the first screening of contact patients was performed with the Xpert^®^ Carba-R PCR assay (Cepheid, USA).

Whole genome sequencing was performed using an Illumina platform on at least one isolate per patient and environmental site. Sequence reads were analyzed using BioNumerics™ (version 8.1, available at (http://www.applied-maths.com) with default settings, except for the *de-novo* assembly, which was performed using the Unicycler pipeline. Multilocus Sequence Typing (MLST) was determined based on the public scheme available at https://bigsdb.pasteur.fr/klebsiella. For genome comparison, we performed whole genome MLST (wgMLST) using a scheme developed by Applied Maths. Clustering was performed using the categorical-difference coefficient, and a minimum spanning tree for categorical data was built with single and double locus variance priority rules.

### Ethical considerations

The data were obtained during service evaluation and outbreak management activities. According to the Swiss national law, this type of work is exempt from the requirement for approval by the competent research ethics committee.

## Results

### Outbreak description

**Fig. 2:**
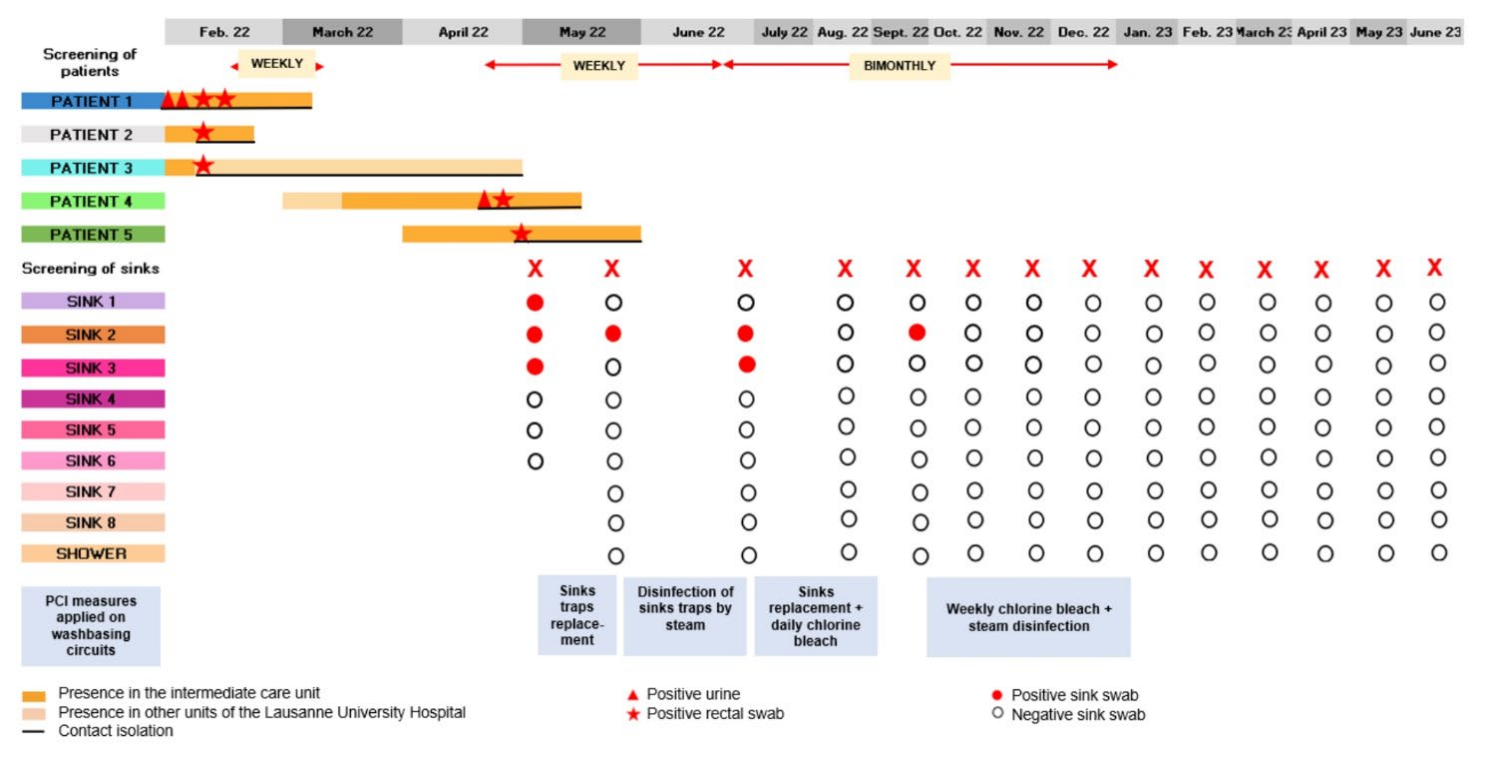
Summary table of hospital stay of NDM-KP positive patients, environmental investigations and interventions on sink traps

In February 2022, the first case of NDM-KP was identified in a urine culture from a patient who had been hospitalised for two months in the neurosurgical intermediate care unit. Between February and May 2022, four more patients were identified as newly colonised with NDM-KP, including a patient with a *bla*NDM -positive PCR but a negative culture. All five patients had been hospitalised in the neurosurgical intermediate care unit, and none had been hospitalised abroad. The outbreak unfolded in a biphasic way: three patients were diagnosed in February 2022 (one with a positive urine culture and two contacts with positive rectal samples), and two in April and May 2022 (Fig. 2). The second phase included a new case with a positive urine culture and a contact with a positive rectal sample, both admitted after the first three cases had been discharged. Among the five cases, four were identified during their stay in the neurosurgical intermediate care unit, and one was diagnosed nine days after being transfered to a rehabilitation unit. The time from hospital admission to NDM-KP identification ranged from 13 to 65 days and the duration of hospitalisation in the intermediate care unit spanned from 23 to 99 days. For all patients, acquisition was considered linked to the stay in the intermediate care unit. No digestive decolonisation regimen was proposed to the patients, due to the lack of evidence on the efficacy of such strategies for carbapenemase producing bacteria [14, 18]. 

### Initial IPC measures

According to Swiss national guidelines [17], IPC measures were applied promptly after the detection of the first NDM-KP case. Upon identification of the fourth and fifth cases in April and May 2022, the IPC team assessed practices using a national standardized method which revealed suboptimal adherence to hand hygiene (51%) and frequent misuse of gloves (no indication for use, not properly changed). Additionally, an improper usage of washbasins during patient care was observed, including the direct spillage in sinks of water used for washing and the use of handwashing basins for cleaning medical devices such as tracheostomy tubes. Following these observations, education rounds to reinforce hand hygiene, and material and environment disinfection were implemented. Weekly screening of inpatients in the unit and enhanced environmental cleaning were also pursued.

### Environmental investigations

In view of the new cases found during the second phase of the outbreak, environmental samples from all P-traps and the shower drain were performed in search of a persistent reservoir. Among the six sink traps sampled, three (50%) were positive for NDM-KP. Follow-up testing showed persistent colonisation by the epidemic strain in two sinks in June and in one sink in September 2022 (Fig. 2).

### Genomic typing

**Fig. 3:**
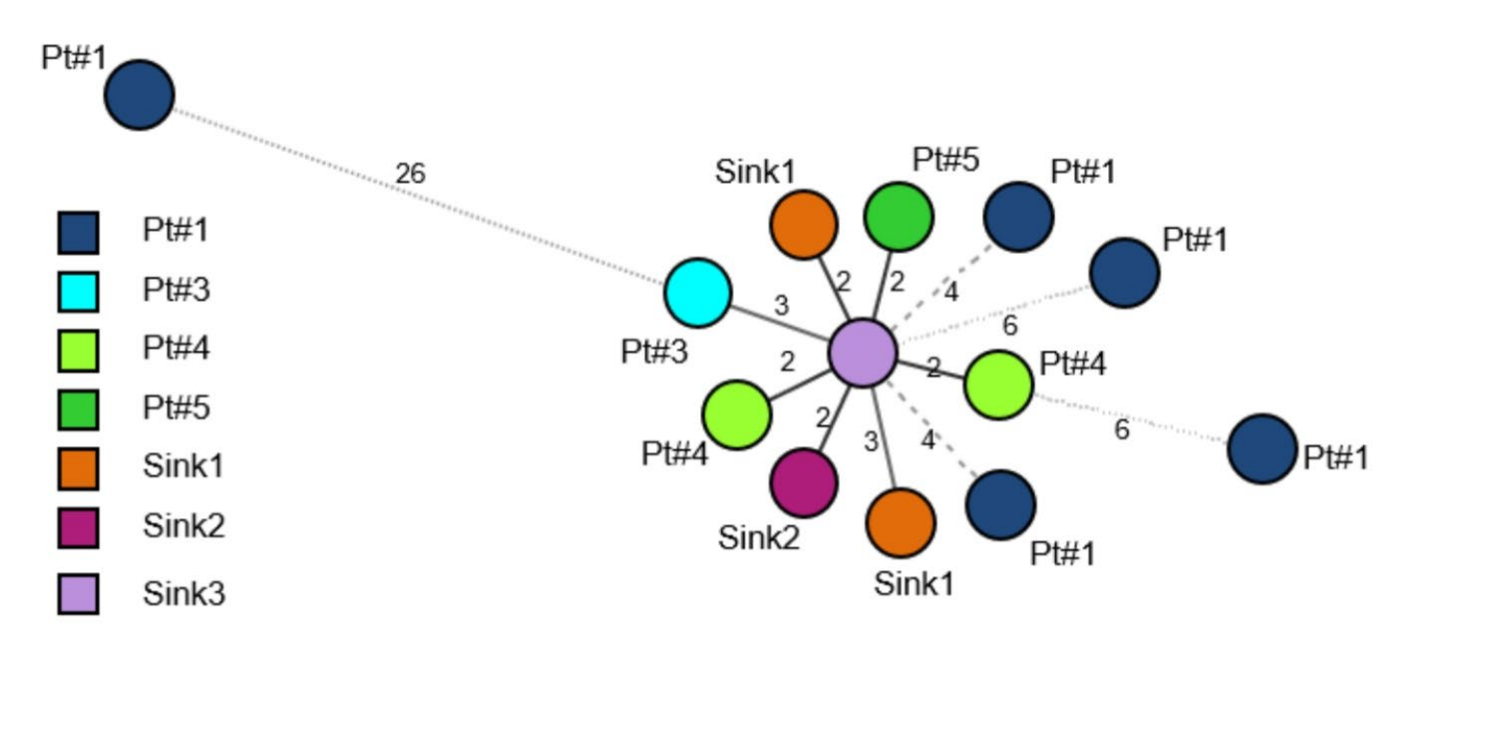
Minimum spanning tree of ST268 strains. Each circle represents one isolate. The distance between the circles represents the number of different loci

Genomic typing was performed on NDM-KP isolates successfully cultured from four out of five patients and from all environmental samples. All isolates belonged to MLST ST-268, and their genomes were closely related (0 to 6 loci differences) based on wgMLST (Fig. 3). One of the 5 isolates of case 1 (39087) showed a 26 loci difference, possibly reflecting this patient’s bacterial flora diversity. Moreover, the strains isolated from the P-traps were genetically very close, suggesting cross-transmission.

### Enhanced IPC measures

Considering the environmental and genomic evidence suggesting a wastewater-associated reservoir, we implemented “water-free” patient care, a strategy routinely utilized in our ICU. In addition, we replaced the three NDM-KP-positive P-traps (Fig. 2). Follow-up sampling performed two weeks later showed persistent colonisation of a sink trap by the same isolate. At this point, we initiated weekly steam disinfection of all sink traps, performed by the cleaning team. Despite these measures, follow-up sampling one month later revealed persistent colonisation of two P-traps. Given the unsuccessful eradication of the environmental reservoir with the aforementioned measures, we replaced the whole washbasin circuit of colonised sinks and implemented daily disinfection by chlorine bleach (one litre per day per colonised sink followed by sink condemnation during at least 30 min) for two consecutive weeks. New environmental sampling was negative two weeks after the end of the disinfection procedure but turned out positive once again in a previously colonized sink, a month later. Due to the persistent reservoir, we implemented a combined disinfection with chlorine bleach and steam over 3 months and pursued monthly environmental sampling of all P-traps for another 5 months. Follow-up samples of all sink traps and the shower drain, performed until May 2023, remained negative. CPE screening of all inpatients in the unit was continued on a weekly and then bimonthly basis, until December 2022. To date (July 2024) no additional epidemiologically or genomically related NDM-KP-positive patients have been identified. Moreover, no NDM-producing bacteria were found in other environmental samples within our hospital.

## Discussion

We report the successful control of an environmental reservoir of NDM-KP in a low-incidence setting, over an eight-month period. During the investigation of this outbreak, WGS analysis allowed us to detect clonal relationships between clinical and environmental isolates, and to identify a probable transmission pathway contributing to the persistence of the epidemic strain in the unit.

Many reports have described outbreaks of MDRO linked to the hospital wastewater systems [10, 11, 15, 19–24]. Highly discriminatory genomic methods, such as WGS, now allow for the confirmation of the key role that wastewater drains play as reservoir [8, 9, 23, 25–27]. Such transmissions have been mainly reported in ICU and hematology-oncology wards, predominantly affecting immunosuppressed patients exposed to several medical devices [6]. In Switzerland, carbapenem resistance remain rare, although numbers are increasing steadily, mirroring the situation in neighbouring countries. *Klebsiella pneumoniae* producing NDM, oxacillinas*e* or *Klebsiella pneumoniae* carbapenemase and *Escherichia coli* producing oxacillinas*e* or NDM are the most frequently observed CPE strains [2, 28, 29]. Recently, Catho et al. [30] published a report on a long-term outbreak of *Pseudomonas aeruginosa* producing Verona integron-encoded metallo-*β*-lactamase carbapenemase, which was genetically related to the hospital building’s wastewater in an intensive care unit in Geneva. This highlights the rising threat of carbapenemase-producing microorganisms in Swiss hospitals.

The biofilm present within the hospital wastewater system creates a particularly conducive environment for the development of a resistant microbiome. Indeed, the repeated exposure to various biological fluids from patients [30] or to antibiotic treatments poured into washbasins encourages the establishment and selection of antibiotic-resistant micro-organisms and therefore the potential horizontal transfer of resistance genes between species. Nevertheless, factors affecting MDRO establishment in the hospital wastewater environment are complex. In the case of carbapenemase-producing *K. pneumoniae*, Park et al. [7] showed that positive patients can seed the wastewater environment in at least 6% of opportunities. Additionally, environmental sites that become colonised are more likely to remain positive, which is congruent with our experience.

Several routes of transmission between patients and aqueous reservoirs have been reported, including retro-contamination via splashes of the healthcare environment [31, 32], healthcare equipment and medical devices [27]. The routes can lead to patient contamination through direct or indirect contact. Sub-optimal design or misuse of sinks may contribute to the dissemination of microorganisms colonizing the washbasin circuit [33, 34]. In our neurosurgical intermediate care unit, washbasins lack devices designed to minimise splashing, such as taps that do not flow directly into the drain and physical barriers that separate the adjacent area. Nevertheless, all waterpoints are located more than one meter far from patients’ beds, and patients’ clean items are stored far from the sink environment. However, on-site observations revealed several improper uses of washbasins during patient care and suboptimal hand hygiene adherence, which may have contributed to cross-transmission. The role of hand-carried transmission is difficult to evaluate and remains undefined, largely due to the likely contribution of multimodal factors. Nonetheless, several studies have reported a significant reduction in transmission following enhancements in hand hygiene compliance [35]. 

Therefore, bundled approaches that aims to prevent the seeding of hospital drains with MDROs, by avoiding patient fluid exposition, eradicating colonized reservoirs, and interrupting cross-transmissions between patients and their water environment, have been proposed and led to a more or less satisfactory control of epidemic situations [6]. Modifying or renovating washbasin pipelines, including the revaluation of pipe material (e.g., copper) and the installation of self-cleaning sinks using vibration/heat/ultrasonic [28], can be challenging mostly due to cost and architectural constraints. Other feasible but difficult-to-implement measures include anti-splashing barriers [28, 36] and safe sink practices, such as hand hygiene-dedicated washbasins and disposal of human waste (including water in contact with patients or medical equipment) in a washer-disinfector [34]. Importantly, waterless patient care significantly contributes to the prevention of water environment-to-patient cross-transmission, but is not always accepted by healthcare providers [14, 30, 37].

Regarding the eradication of environmental reservoirs, various drain treatment protocols, primarily involving chemical agents (such as bleach and hydrogen peroxide) [38] or thermal disinfection, have been documented [39]. However, these methods have shown limited, if any, lasting effect on decolonizing MDROs from drain system. The presence of microbial biofilm in sink traps hinders eradication of microorganisms and may warrant the replacement of the whole washbasin circuit. Nevertheless, P-traps replacement alone is often insufficient, as biofilm-derived re-colonization can occur from distal (unchanged) components of the drain system [11]. In our case, partial replacement of the drainage circuit by changing the traps only was not successful, even when combined with weekly steam disinfection. Only the combination of a complete washbasin circuit replacement with bleach and heat disinfection allowed the sustained control of the environmental reservoir, after a 12-month follow-up. The persistent contamination of P-traps after initial replacement highlights the importance of replacing the connecting pieces, while working closely with the plumbing department. More data from prospective studies comparing different replacement and disinfection protocols are required.

Only a few studies reporting success stated the duration of follow-up after the intervention, which ranged, from 2 months to 3.5 years [23, 36, 40, 41]. In our situation, we continued microbiological screening of P-traps for 12 months after the identification of the last positive patient and 5 months after the completion of the sink’s disinfection protocol, providing proof for a successful and persistent control of the outbreak. Nevertheless, a possible limitation is the potential undersampling of the aqueous environment, as sampling only sink and shower traps may not suffice to conclusively demonstrate the absence of NDM genes.

The unicentric and observational character of this report represents a key limitation. Moreover, the genomic analysis was limited to the complete sequencing of strains but did not include a plasmid analysis to determine if the NDM plasmids were related. Finally, we used a multimodal approach to control the NDM-KP outbreak and therefore, we were not able to assess each IPC measure separately. Nevertheless, we provide a structured and detailed description of an effective bundle of measures which can be reproduced in other settings.

## Conclusion

In conclusion, our comprehensive investigation, spanning several months and utilizing both patient and environmental screenings alongside WGS analysis, was pivotal in the successful control of NDM-KP. This outcome was achieved through the implementation of a multimodal IPC strategy, underscored by waterless patient care and the meticulous replacement and disinfection of P-traps and connecting pieces. Crucially, the success of this endeavor was further enhanced by effective collaboration with plumbing and cleaning teams, demonstrating that in low-incidence settings, proactive and coordinated efforts can substantially mitigate and even completely eliminate the transmission of CPE. This case study underscores the imperative for ongoing vigilance, interdisciplinary collaboration, and innovation in IPC measures to address the evolving challenge of MDROs in healthcare environments globally.

## Data Availability

No datasets were generated or analysed during the current study.

## Abbreviations

CPE: Carbapenemase-producing Enterobacteriacea
NDM-KP: Klebsiella pneumoniae producing New Delhi metallo-beta-lactamase carbapenemase
WGS: Whole genome sequencing
wgMLST: Whole genome multilocus sequence typing
IPC: Infection prevention and control
PCR: Polymerase chain reaction
ICU: Intensive care unit
MDRO: Multidrug resistant organism

## References

[1] European Centre for Disease Prevention and Control. Carbapenem-resistant Enterobacteriaceae, second update – 26 September 2019. Volume ECDC. Stockholm; 2019.

[2] Federal Office of Public Health and Federal Food Safety and Veterinary Office. Swiss Antibiotic Resistance Report 2022. Usage of Antibiotics and Occurrence of Antibiotic Resistance in Switzerland. November 2022.

[3] Jamal AJ, Mataseje LF, Brown KA, Katz K, Johnstone J, Muller MP, et al. Carbapenemase-producing enterobacterales in hospital drains in Southern Ontario, Canada. J Hosp Infect. 2020;106(4):820–7. 32916210 10.1016/j.jhin.2020.09.007

[4] Lemarie C, Legeay C, Kouatchet A, Mahieu R, Lasocki S, Holecska P, et al. High prevalence of contamination of sink drains with carbapenemase-producing Enterobacteriaceae in 4 intensive care units apart from any epidemic context. Am J Infect Control. 2020;48(2):230–2. 31495643 10.1016/j.ajic.2019.08.007

[5] Regev-Yochay G, Smollan G, Tal I, Pinas Zade N, Haviv Y, Nudelman V, et al. Sink traps as the source of transmission of OXA-48-producing Serratia marcescens in an intensive care unit. Infect Control Hosp Epidemiol. 2018;39(11):1307–15. 30284524 10.1017/ice.2018.235

[6] Kearney A, Boyle MA, Curley GF, Humphreys H. Preventing infections caused by carbapenemase-producing bacteria in the intensive care unit - think about the sink. J Crit Care. 2021;66:52–9. 34438134 10.1016/j.jcrc.2021.07.023

[7] Park SC, Parikh H, Vegesana K, Stoesser N, Barry KE, Kotay SM et al. Risk factors Associated with Carbapenemase-Producing Enterobacterales (CPE) positivity in the Hospital Wastewater Environment. Appl Environ Microbiol. 2020;86(24). 10.1128/AEM.01715-20 PMC7688209 32917755

[8] Jung J, Choi HS, Lee JY, Ryu SH, Kim SK, Hong MJ, et al. Outbreak of carbapenemase-producing Enterobacteriaceae associated with a contaminated water dispenser and sink drains in the cardiology units of a Korean hospital. J Hosp Infect. 2020;104(4):476–83. 31785319 10.1016/j.jhin.2019.11.015

[9] Leitner E, Zarfel G, Luxner J, Herzog K, Pekard-Amenitsch S, Hoenigl M, et al. Contaminated handwashing sinks as the source of a clonal outbreak of KPC-2-producing Klebsiella oxytoca on a hematology ward. Antimicrob Agents Chemother. 2015;59(1):714–6. 25348541 10.1128/AAC.04306-14 PMC4291428

[10] Vergara-Lopez S, Dominguez MC, Conejo MC, Pascual A, Rodriguez-Bano J. Wastewater drainage system as an occult reservoir in a protracted clonal outbreak due to metallo-beta-lactamase-producing Klebsiella oxytoca. Clin Microbiol Infect. 2013;19(11):E490–8. 23829434 10.1111/1469-0691.12288

[11] Carling PC. Wastewater drains: epidemiology and interventions in 23 carbapenem-resistant organism outbreaks. Infect Control Hosp Epidemiol. 2018;39(8):972–9. 29950189 10.1017/ice.2018.138

[12] Hamerlinck H, Aerssens A, Boelens J, Dehaene A, McMahon M, Messiaen AS, et al. Sanitary installations and wastewater plumbing as reservoir for the long-term circulation and transmission of carbapenemase producing Citrobacter freundii clones in a hospital setting. Antimicrob Resist Infect Control. 2023;12(1):58. 37337245 10.1186/s13756-023-01261-9 PMC10280848

[13] Clarivet B, Grau D, Jumas-Bilak E, Jean-Pierre H, Pantel A, Parer S et al. Persisting transmission of carbapenemase-producing Klebsiella pneumoniae due to an environmental reservoir in a university hospital, France, 2012 to 2014. Euro Surveill. 2016;21(17). 10.2807/1560-7917.ES.2016.21.17.30213 27168586

[14] Catho G, Huttner BD. Strategies for the eradication of extended-spectrum beta-lactamase or carbapenemase-producing Enterobacteriaceae intestinal carriage. Expert Rev Anti Infect Ther. 2019;17(8):557–69. 31313610 10.1080/14787210.2019.1645007

[15] Decraene V, Phan HTT, George R, Wyllie DH, Akinremi O, Aiken Z et al. A large, refractory nosocomial outbreak of Klebsiella pneumoniae carbapenemase-producing Escherichia coli demonstrates Carbapenemase Gene outbreaks Involving Sink sites require novel approaches to infection control. Antimicrob Agents Chemother. 2018;62(12). 10.1128/AAC.01689-18 PMC6256751 30249685

[16] Fankhauser C, Zingg W, Francois P, Dharan S, Schrenzel J, Pittet D, et al. Surveillance of extended-spectrum-beta-lactamase-producing Enterobacteriaceae in a Swiss Tertiary Care Hospital. Swiss Med Wkly. 2009;139(51–52):747–51. 19924582 10.4414/smw.2009.12918

[17] Vuichard-Gysin DBN, Tschudin-Sutter S, Senn L, Kuster S, Metsini A, Eder M, Widmer A. Gestion Des épidémies dues à des bactéries multirésistantes (BMR). Centre national de prévention des infections(Swissnoso); 2021.

[18] Huttner BD, de Lastours V, Wassenberg M, Maharshak N, Mauris A, Galperine T, et al. A 5-day course of oral antibiotics followed by faecal transplantation to eradicate carriage of multidrug-resistant Enterobacteriaceae: a randomized clinical trial. Clin Microbiol Infect. 2019;25(7):830–8. 30616014 10.1016/j.cmi.2018.12.009

[19] Kanamori H, Weber DJ, Rutala WA. Healthcare Outbreaks Associated with a Water Reservoir and infection Prevention Strategies. Clin Infect Dis. 2016;62(11):1423–35. 26936670 10.1093/cid/ciw122

[20] Exner M, Kramer A, Lajoie L, Gebel J, Engelhart S, Hartemann P. Prevention and control of health care-associated waterborne infections in health care facilities. Am J Infect Control. 2005;33(5 Suppl 1):S26–40. 15940114 10.1016/j.ajic.2005.04.002

[21] Chia PY, Sengupta S, Kukreja A, S SLP, Ng OT, Marimuthu K. The role of hospital environment in transmissions of multidrug-resistant gram-negative organisms. Antimicrob Resist Infect Control. 2020;9(1):29. 32046775 10.1186/s13756-020-0685-1 PMC7014667

[22] Lewis SS, Smith BA, Sickbert-Bennett EE, Weber DJ. Water as a source for colonization and infection with multidrug-resistant pathogens: focus on sinks. Infect Control Hosp Epidemiol. 2018;39(12):1463–6. 30526717 10.1017/ice.2018.273

[23] Kizny Gordon AE, Mathers AJ, Cheong EYL, Gottlieb T, Kotay S, Walker AS, et al. The Hospital Water Environment as a Reservoir for Carbapenem-resistant organisms causing hospital-acquired Infections-A systematic review of the literature. Clin Infect Dis. 2017;64(10):1435–44. 28200000 10.1093/cid/cix132

[24] Kotsanas D, Wijesooriya WR, Korman TM, Gillespie EE, Wright L, Snook K, et al. Down the drain: carbapenem-resistant bacteria in intensive care unit patients and handwashing sinks. Med J Aust. 2013;198(5):267–9. 23496403 10.5694/mja12.11757

[25] Caggiano P, Bertone E, Cocchini G. Same action in different spatial locations induces selective modulation of body metric representation. Exp Brain Res. 2021;239(8):2509–18. 34142190 10.1007/s00221-021-06135-3

[26] Nurjadi D, Kocer K, Chanthalangsy Q, Klein S, Heeg K, Boutin S. New Delhi Metallo-Beta-Lactamase facilitates the emergence of Cefiderocol Resistance in Enterobacter cloacae. Antimicrob Agents Chemother. 2022;66(2):e0201121. 34871093 10.1128/aac.02011-21 PMC8846454

[27] Grabowski M, Lobo JM, Gunnell B, Enfield K, Carpenter R, Barnes L, et al. Characterizations of handwashing sink activities in a single hospital medical intensive care unit. J Hosp Infect. 2018;100(3):e115–22. 29738784 10.1016/j.jhin.2018.04.025

[28] Mathers AJ, Vegesana K, German Mesner I, Barry KE, Pannone A, Baumann J, et al. Intensive Care Unit Wastewater Interventions to prevent transmission of multispecies Klebsiella pneumoniae carbapenemase-producing organisms. Clin Infect Dis. 2018;67(2):171–8. 29409044 10.1093/cid/ciy052

[29] [ https://www.anresis.ch/

[30] Catho G, Martischang R, Boroli F, Chraiti MN, Martin Y, Koyluk Tomsuk Z, et al. Outbreak of Pseudomonas aeruginosa producing VIM carbapenemase in an intensive care unit and its termination by implementation of waterless patient care. Crit Care. 2021;25(1):301. 34412676 10.1186/s13054-021-03726-y PMC8376114

[31] Kotay SM, Parikh HI, Barry K, Gweon HS, Guilford W, Carroll J, et al. Nutrients influence the dynamics of Klebsiella pneumoniae carbapenemase producing enterobacterales in transplanted hospital sinks. Water Res. 2020;176:115707. 32224328 10.1016/j.watres.2020.115707

[32] Hota S, Hirji Z, Stockton K, Lemieux C, Dedier H, Wolfaardt G, et al. Outbreak of multidrug-resistant Pseudomonas aeruginosa colonization and infection secondary to imperfect intensive care unit room design. Infect Control Hosp Epidemiol. 2009;30(1):25–33. 19046054 10.1086/592700

[33] Gestrich SA, Jencson AL, Cadnum JL, Livingston SH, Wilson BM, Donskey CJ. A multicenter investigation to characterize the risk for pathogen transmission from healthcare facility sinks. Infect Control Hosp Epidemiol. 2018;39(12):1467–9. 30526714 10.1017/ice.2018.191

[34] Parkes LO, Hota SS. Sink-related outbreaks and mitigation strategies in Healthcare Facilities. Curr Infect Dis Rep. 2018;20(10):42. 30128678 10.1007/s11908-018-0648-3

[35] Decker BK, Palmore TN. The role of water in healthcare-associated infections. Curr Opin Infect Dis. 2013;26(4):345–51. 23806897 10.1097/QCO.0b013e3283630adf PMC5583640

[36] Pirzadian J, ‘t Voor. Holt AF, Hossain M, Klaassen CHW, de Goeij I, Koene H, Limiting spread of VIM-positive Pseudomonas aeruginosa from colonized sink drains in a tertiary care hospital: A before-and-after study. PLoS One. 2023;18(3):e0282090. 10.1371/journal.pone.0282090 PMC10038242 36961784

[37] Hopman J, Tostmann A, Wertheim H, Bos M, Kolwijck E, Akkermans R, et al. Reduced rate of intensive care unit acquired gram-negative bacilli after removal of sinks and introduction of ‘water-free’ patient care. Antimicrob Resist Infect Control. 2017;6:59. 28616203 10.1186/s13756-017-0213-0 PMC5466749

[38] Buchan BW, Arvan JA, Graham MB, Tarima S, Faron ML, Nanchal R, et al. Effectiveness of a hydrogen peroxide foam against bleach for the disinfection of sink drains. Infect Control Hosp Epidemiol. 2019;40(6):724–6. 30992089 10.1017/ice.2019.72

[39] De Geyter D, Blommaert L, Verbraeken N, Sevenois M, Huyghens L, Martini H, et al. The sink as a potential source of transmission of carbapenemase-producing Enterobacteriaceae in the intensive care unit. Antimicrob Resist Infect Control. 2017;6:24. 28239453 10.1186/s13756-017-0182-3 PMC5314675

[40] Smolders D, Hendriks B, Rogiers P, Mul M, Gordts B. Acetic acid as a decontamination method for ICU sink drains colonized by carbapenemase-producing Enterobacteriaceae and its effect on CPE infections. J Hosp Infect. 2019;102(1):82–8. 30579969 10.1016/j.jhin.2018.12.009

[41] Nurjadi D, Scherrer M, Frank U, Mutters NT, Heininger A, Spath I, et al. Genomic investigation and successful Containment of an intermittent common source outbreak of OXA-48-Producing Enterobacter cloacae related to Hospital Shower drains. Microbiol Spectr. 2021;9(3):e0138021. 34817232 10.1128/Spectrum.01380-21 PMC8612159

